# Galectin-8, cytokines, and the storm

**DOI:** 10.1042/BST20200677

**Published:** 2021-01-11

**Authors:** Yehiel Zick

**Affiliations:** Department of Molecular Cell Biology, Weizmann Institute of Science, Rehovot 76100, Israel

**Keywords:** cytokine storm, cytokines, galectin-8

## Abstract

Galectin-8 (Gal-8) belongs to a family of animal lectins that modulate cell adhesion, cell proliferation, apoptosis, and immune responses. Recent studies have shown that mammalian Gal-8 induces in an autocrine and paracrine manner, the expression and secretion of cytokines and chemokines such as RANKL, IL-6, IL-1β, SDF-1, and MCP-1. This involves Gal-8 binding to receptor complexes that include MRC2/uPAR/LRP1, integrins, and CD44. Receptors ligation triggers FAK, ERK, Akt, and the JNK signaling pathways, leading to induction of NF-κB that promotes cytokine expression. Indeed, immune-competent Gal-8 knockout (KO) mice express systemic lower levels of cytokines and chemokines while the opposite is true for Gal-8 transgenic animals. Cytokine and chemokine secretion, induced by Gal-8, promotes the migration of cancer cells toward cells expressing this lectin. Accordingly, Gal-8 KO mice experience reduced tumor size and smaller and fewer metastatic lesions when injected with cancer cells. These observations suggest the existence of a ‘vicious cycle’ whereby Gal-8 expression and secretion promotes the secretion of cytokines and chemokines that further promote Gal-8 expression. This ‘vicious cycle’ could enhance the development of a ‘cytokine storm’ which is a key contributor to the poor prognosis of COVID-19 patients.

## Introduction

Galectin-8 (Gal-8) belongs to a family of animal lectins that bind different glycoconjugates [1–3]. Galectins are divided into three groups: (i) prototype galectins (Gal-1, -2, -5, -7, -10, -11, -13 to -16), having one carbohydrate-recognition domain (CRD); (ii) tandem-repeat type galectins (Gal-4, -6, -8, -9, and-12) that have two different CRDs joined by a linker peptide; and (iii) a chimera-type Gal-3 that has a single CRD joined to an N-terminal non-lectin domain [1–4]. The Gal-8 gene (*LGALS8*) encodes at least four isoforms that differ in the size of their linker peptide that ranges from 24 to 74 amino acids. The two CRDs spaced at different distances presumabely bind different spatially oriented carbohydrates that affect the function of Gal-8 [5]. Galectins including Gal-8 lack an N-terminal signal sequence to direct them through to the ER, therefore, they are secreted by an atypical secretion mechanism [6]. It might involve their direct translocation across membranes; export via lysosomes or endosomes; release in exosomes or export via micro-vesicles [6]. As a secreted protein Gal-8 is present in body fluids (e.g. synovial fluids of RA patients (25–60 nM) [7] or serum of breast (4.7–233.2 ng/ml) and colon (5.6–178.2 ng/ml) cancer patients [8]). Extracellular Gal-8 promotes cell adhesion upon binding to cell adhesion molecules such as integrins [9–13], CD44 [7], CD166 [14], and Podoplanin [15].

Although the extracellular carbohydrate-binding activities of galectins became their defining feature [1–3,13,16,17], intracellular galectins accomplish various functions by interacting with multiple ligands using CRD-dependent and -independent interactions [18,19]. Gal-8 exerts intracellular functions by labeling pathogen-invaded vacuoles for their destruction by autophagy [20,21]. Gal-8 inhibits mTOR signaling during endomembrane perturbations as a result of lysosomal damage [22], while binding of Gal-8 to farnesylated K-Ras4B inhibits Ras activation [23]. These observations implicate intracellular Gal-8 in signaling networks involved in homeostatic repair, removal, and replacement of damaged endomembranes.

Galectins, including Gal-8, emerge as key regulators of primary tumor growth and metastasis [24–31]. Amplification of *LGALS8* and increased Gal-8 expression is observed in various cancerous tissues [32–35] including breast, prostate, and lung [32–34,36–38] and is often associated with poor prognosis [32]. Tumor invasiveness and metastatic dissemination are regulated by immunomodulators [39], including cytokines and chemokines. These are well-known chemo-attractants that stimulate migration of malignant cells towards their metastatic niche [25,39,40]; serving as maintenance and survival factors of cancer cells [41]. Cytokine and chemokine expression is governed by signaling pathways [42] triggered upon ligation of receptors that include Toll-like receptors (TLRs) [43]; tumor necrosis factor (TNF-R), and interleukin-1 (IL-1R). This leads to activation of transcription factors including nuclear factor-κB (NF-κB) that plays a key role in cytokine production [44–46]. TLR, IL-1R, and TNF-R signaling to NF-κB converge on a common IκB kinase complex that phosphorylates the NF-κB inhibitory protein IκBα leading to its degradation and activation of p100 and p105, the precursors of NFκB1 and NFκB2, respectively [47].

Mammalian galectins are important mediators of adaptive and innate immune responses [26,48]. As such they are implicated in immune regulatory cancer networks that involve cytokine and chemokine production and action [8,24,49]. Yet, the direct effects of galectins including Gal-8 on cytokine/chemokine expression in non-immune cells remain incompletely understood. Even less studied are the reciprocal effects of cytokines and chemokines on the expression, secretion, and function of galectins. These issues are the subject of the current review.

## Effects of Gal-8 on cytokine and chemokine expression

Galectins are known mediators involved in the recruitment of inflammatory cells to target tissues [50–55]. Given the central role of cytokines and chemokines in this process, galectins were implicated in the regulation of cytokine/chemokine expression and secretion; inhibition of cytokine diffusion through the extracellular matrix and modulation of cytokine signaling, as discussed below.

Similar to other galectins, Gal-8 affects both adaptive and innate immune responses [52]. Gal-8 targets cytokine-receptor interactions, as well as focal adhesion and TNF signaling [56] in bone-marrow-derived mouse dendritic cells (BMDCs) that induces secretion of IL-3, IL-2, IL-6, IL-13, TNF-α, MCP-1, MCP-5, G-CSF, and GM-CSF [57]. Gal-8 activates splenic B cell proliferation, and promotes the production of IL-6 and IL-10 [58]. Gal-8-induced proliferation of naïve CD4+ T cells is accompanied by increased expression of IL-2, IFN-γ, and IL-4 [52]. Gal-8's effects on primary CD4^+^ T cells are mediated by the CD45 P-Tyr phosphatase activity and involve activation of ZAP-70 and the ERK1/2 signaling pathways [59].

Of note, Gal-8 induces cell death and inhibits the proliferation of stimulated T cells involved in immune responses. In a model of autoimmune uveitis, Gal-8 administration increases the number of CTLA-4^+^IL-10^+^CD103^+^ Treg cells as well as Th2 cells and impairs the production of inflammatory cytokines by retinal Th1 and Th17 cells [60]. This dual function of Gal-8 in stimulating or inhibiting cytokine production in naïve vs. stimulated immune cells could be rationalized by at least two mechanisms: It could be attributed to the differential glycosylation profile exhibited by naïve vs. activated cells, that express selective Gal-8 binding partners that dictate the intracellular signaling and the outcome response [52]. Alternatively, Gal-8, similar to Gal-1 and Gal-3, could form heterodimers with chemokines primarily involved in later stages of inflammation to inhibit their activity [61] (*vide infra*).

Reports concerning the effects of galectins on cytokine and chemokine expression in non-immune cells are less abundant [62–66]. Oxidized Gal-1 that lost lectin property gained new activity to induce expression of MMP9 and inflammatory cytokines through activation of ERK signaling in a sugar-independent manner [67]. Similarly, direct interaction of intracellular Gal-9 with stimulator of interferon genes (STING) promotes ubiquitination and degradation of STING [68], thus leading to enhanced cytokine production. These results indicate that galectins acting intracellularly might regulate cytokine production. Given that STING is not glycosylated, these findings further implicate protein–protein interactions between Gal-9 and STING.

Gal-8 was shown to promote in primary osteoblasts the expression and secretion of the cytokine-receptor activator of NF-κB ligand (RANKL) [69]. This involved Gal-8 binding to receptors that positively (uPAR and MRC2) and negatively (LRP1) mediated differentiation into osteoclasts of bone-marrow cells co-cultured with Gal-8-treated osteoblasts [69]. Treatment of osteoblasts with Gal-8 significantly increases 5–60-fold the mRNA levels of additional chemokines and cytokines including SDF-1, TNF-α, IL-1β, MCP-1, IP10, and IL-6 [70]. The stimulatory effects of Gal-8 on cytokine expression and secretion are a general phenomenon observed in many cell types and tissues including liver spleen and lungs [70], suggesting that Gal-8 regulates chemokines expression in non-immune cells.

## Induction of cytokine expression by Gal-8 independent of its sugar-binding properties

Gal-8 acts as an extracellular ligand that activates signaling pathways both by protein–sugar and protein–protein interactions [71]. Indeed, recombinant Gal-8 promotes RANKL expression in primary cultured osteoblasts in a sugar-dependent manner, because a Gal-8 mutant W2Y (W85Y and W248Y) that lacks sugar-binding activity fails to reproduce these effects [69,70]. Similarly, the sugar analog TDG partially inhibits the stimulatory effects of recombinant Gal-8 on RANKL expression. In contrast, the recombinant Gal-8-W2Y mutant is almost perfectly capable of inducing the expression of SDF-1 and MCP-1, suggesting that their expression is mediated through Gal-8 binding to cell surface receptors in a sugar-independent manner [69,70].

Dual recognition by animal lectins of both glycan and aglycon moieties is well established [72]. Protein–protein interactions constitute part of the cytostatic effects of Gal-1 [73]. Similarly, intracellular Gal-3 interacts with a protein termed Alix in a sugar-independent manner [74], whereas binding of extracellular Gal-3 to SDF-1 involves regions independent of its carbohydrate-binding domain [61]. The ability of Gal-8 to engage in protein–protein interactions is well established [72,75,76]. Protein–protein interactions mediate the binding of intracellular Gal-8 to NDP52, the autophagy cargo receptor [77]. Interestingly NDP52-binding to Gal-8 C-terminal CRD is on its convex side opposite to the galactose-binding concave side; thus Gal-8 can bind both to carbohydrate and target protein simultaneously [77].

## Molecular mechanisms underlying Gal-8 induction of cytokine expression

Gal-8-induced expression of cytokines such as RANKL is mediated through Gal-8 binding to receptor complexes that include uPAR, MRC2, and LRP1 [69]. uPAR co-immunoprecipitates with integrins and integrin-associated signaling molecules such as FAK and Src family kinases (reviewed in [78]) to modulate the affinity of β_1_, β_2_, and β_3_ integrins [79]. Integrins, including β_1_, αM, α_3_β_1_, and α_6_β_1_, as well as other ECM proteins, also serve as binding partners to Gal-8 that functions as a matricellular protein [9,11,13,80]. Complex formation between extracellular Gal-8 and integrins triggers integrin-mediated signaling cascades such as Tyr phosphorylation of FAK and paxillin, and a robust and sustained activation of the ERK and PI3K pathways [9,10,81,82]. Hence, the interaction of Gal-8 with a complex of the uPAR/LRP1/MRC2 that binds integrins could be the mechanism underlying the transcription of RANKL and other cytokines in response to Gal-8. In contrast, Gal-8 mediates its effects on SDF-1 expression through binding to LRP1 and uPAR, but not MRC2, suggesting that ligation of extracellular Gal-8 by different receptor complexes triggers expression of different sets of cytokines. This results in differential activation of downstream signaling pathways. While the effects of Gal-8 on RANKL gene expression are mediated by the ERK signaling pathway [69], JNK mediates Gal-8's effect on SDF-1 [70]. Activations of ERK leads to sustained activation of the NF-κB pathway [83] whereas activation of JNK induces the accumulation of beta-TrCP that mediates ubiquitination and degradation of phosphorylated IkKβ followed by proteasome-dependent degradation of IkB that results in activation of the NF-κB pathway [84].

The above results are supported by other studies that demonstrate a role for Gal-8 in the activation of NF-κB. Treatment of HMEC-1 cells with Gal-8 produces many cytokines in a process that requires activation of NFkB [85]. Enhanced cytokine expression mediated by NF-κB is also observed in OA chondrocytes treated with Gal-8 [86]. Stimulated NF-κB activity in osteoblasts treated with Gal-8 is accompanied by 3–4-fold increased phosphorylation (activation) of IKKα/β and a corresponding reduction in IκB, the downstream target of IKKβ and the upstream activator of NF-κB.

## Alterations in cytokine/chemokine expression in Gal-8-transgenic (Tg) and knockout (KO)-mice

The physiological effects of Gal-8 on cytokines/chemokine expression *in vivo* were studied in Gal-8 transgenic (Tg) and KO mice [69,70,87]. As expected, a systemic reduction (80–95%) in mRNA levels of many cytokines and chemokines including RANKL, IP-10, IL-6, IL-1β, TNF-α, MCP-1, and SDF-1 was observed in osteoblasts, long bones, lungs, and spleen derived from Gal-8 KO mice, when compared with WT mice. Gal-8-Tg mice presented a mirror image with a systemic increase in mRNA levels of RANKL, MCP-1, SDF-1, IP-10, IL-6, IL-1β, and TNF-α [70].

## Effects of intracellular Gal-8 on cytokine/chemokine expression and secretion

While the above results establish a role of extracellular Gal-8 as promoter of cytokine/chemokine expression and secretion, the effects of intracellular Gal-8 on this process are less obvious. Most studied is the action of intracellular Gal-8 as a ‘danger signal’ that labels pathogen-invaded vacuoles for their destruction by autophagy [20]. Gal-8 binding to exposed glycans of damaged pathogen-containing endomembranes results in recruitment of NDP52 that engages the autophagic machinery [20,21]. However, the links between the autophagy-promoting effects of intracellular Gal-8 and its stimulatory effects on cytokine expression *in vivo* is largely obscure, and even might be contradictory. Given that autophagy negatively regulates the activation of inflammasomes [88] and given that inflammasomes such as the NLRP3 mediate IL-1β/IL-18 maturation and release [89], it follows that by promoting autophagy, intracellular Gal-8, might in fact inhibit activation of the NLRP3 inflammasome and the formation of at least a subset of cytokines, such as IL-1β.

Intracellular galectins are likely to engage different signaling pathways [18,90]. Indeed, studies already documented direct interactions between intracellular Gal-1 and H-Ras that leads to activation of the latter [91,92]. Similarly, Gal-3 binding to K-Ras, augments its activation and triggers Ras signaling [93]. In contrast, the binding of Gal-8 to farnesylated K-Ras4B inhibits Ras activation because siRNA-mediated depletion of Gal-8 increases K-Ras4B content and ERK1/2 activity in lung and pancreatic carcinoma cells [23].

mTOR is an upstream activator of the NF-κB signaling pathway [89,94]. Intracellular Gal-8 plays a critical role in mTOR inactivation during lysosomal damage. In resting cells Gal-8 is proximal to mTOR, however, following lysosomal damage Gal-8 is more firmly associated with the mTOR regulators Ragulator and RagA/B, whereas its proximity with mTOR and its adaptor Raptor lessens [22,95]. As a result, mTOR is inactivated and desorbs from the lysosomal membrane to the cytosol. Gal-8 exerts these changes by recognizing exposed luminal glycans of the damaged membranes [22,95]. Given that activation of ERK and mTOR stimulate the NF-κB pathway [83,89,94,96–98], inhibition of mTOR and the Ras–MEK–ERK pathway by intracellular Gal-8 is expected to dampen cytokine/chemokine expression and secretion.

Hence, the extracellular vs. intracellular Gal-8 seem to exert opposing effects on cytokine/chemokine expression, from the perspective of the ERK/mTOR pathways. Given, that Gal-8 Tg mice overexpress cytokines and chemokines while Gal-8 KO animals show dampened cytokine expression, it is reasonable to assume that overall, the stimulatory effects of extracellular Gal-8 on cytokine/chemokine expression overcome the putative inhibitory action of its intracellular counterpart.

## Gal-8 cytokines and cancer

Galectins including Gal-8 emerge as key players in the process of cancer growth and metastasis [49,99]. For example, extracellular Gal-8 concentration is elevated in sera of colon and breast cancer patients, where it supports the adhesion of tumor cells to the microvascular lung endothelium [13]. Similarly, marked increases in immunohistochemical Gal-8 expression were observed in malignant breast tissues [100] and papillary thyroid carcinoma [101]. Gal-8 up-regulation was observed during hypopharyngeal and laryngeal tumor progression [102] and was shown to predict postoperative recurrence of patients with localized T1 clear cell renal cell carcinoma [103]. At the molecular level, Gal-8 promotes adhesive interactions between vascular endothelial cells and multiple myeloma cells [104], while binding of lung cancer cells to a complex of Gal-8 and fibronectin promotes metastatic growth of lung adenocarcinoma [38]. Gal-8 interaction with podoplanin-expressing macrophages promote lymphangiogenesis and lymphoinvasion in breast cancer [15]. The above findings implicate Gal-8 as a promoter of tumor growth, which is in line with its action of as a promoter of cytokine expression and secretion.

However, studies also reported on a negative correlation between the expression of Gal-8 and the progression of certain tumor types. Marked decrease in Gal-8 expression was observed in colon, pancreas, liver, skin, and larynx tissue when comparing malignant to normal tissue [100,105,106]. Decreased Gal-8 expression is a strong marker for recurrence in urothelial carcinoma of the bladder [107]. Similarly, low Gal-8 expression is a favorable prognostic biomarker for the survival of patients with gastric cancer [108]. These data implicate an organ-specific regulation of Gal-8 expression upon the malignant transformation of various tissue types [33]. It further implies a delicate balance between the pro- and anti-cancerous roles of Gal-8 (*vide infra*).

The links between cancer and inflammation are also well established. Up to 20% of all cancers arise in association with chronic inflammation, and most, if not all, solid tumors contain inflammatory infiltrates [109]. Recent evidence shows that crucial components of cancer-related inflammation are involved in a co-ordinated system to influence the development of cancer, and immune cells have a broad impact on tumor initiation, growth and progression [110]. Many of these effects are mediated by pro-inflammatory cytokines such as TNFα and IL-6 that are well-known chemo-attractants that stimulate the migration of malignant cells towards their metastatic niche [39].

Chemokine receptors are expressed by different cancer cells [111] and up-regulation of chemokine-receptor pairs (e.g. (Stromal cell-derived factor 1 (SDF-1/CXCL12)/C-X-C chemokine-receptor type 4 (CXCR4)) promotes metastasis [39].

### Gal-8 promotes chemoattraction of cancer cells

Certain effects of galectins [32,49] including Gal-8, on immune regulatory cancer networks were explored. Most relevant are the observations that Gal-8 present in the serum of cancer patients interacts with blood vascular endothelium and promotes secretion to the circulation of MCP-1, IL-6, and G-CSF. This increases the expression of adhesion molecules on the surface of endothelial cells that triggers endothelial–cancer cell interactions [8].

Using prostate cancer cells and naïve osteoblasts as a model system, it was shown that treatment of osteoblasts with Gal-8 increases ∼2 fold cancer cell migration towards these osteoblasts [70]. The enhanced migration of cancer cells was mediated by SDF-1 and MCP-1, secreted by Gal-8-treated osteoblasts. Accordingly, inhibitors of the SDF-1 receptor (CXCR4) or the MCP-1 receptors effectively abolished the stimulatory effects of Gal-8 on cancer cell migration toward osteoblasts [70]. Gal-8-induced chemoattraction, like its effects on cytokine secretion, are sugar-independent. These results conform with the hypothesis that Gal-8 induces cytokine/chemokine secretion from tissues such as osteoblasts, which facilitates cancer cell migration towards naïve target tissues. The effects of Gal-8 reflect those of other galectins. Gal-3 promotes wound re-epithelialization in corneal, intestinal, and skin wounds [112], and Gal-1 accelerates skin wound healing [113]. Gal-1 enhances migration of human monocyte-derived dendritic cells through extracellular matrices [114] and stimulates motility of human umbilical cord blood-derived MSCs via down-regulation of Smad2/3 and up-regulation of NF-κB [115].

### Gal-8 promotes cancer growth and metastasis *in vivo*

Given that cytokines and chemokines play key roles in tumor progression *in vivo* [39] and given that Gal-8 promotes cytokine and chemokine expression, the effects of its depletion on cancer growth and metastasis were studied in mouse models. Injection of breast cancer cells to the mammary gland of Gal-8-KO female mice resulted in the development of significantly smaller tumors than those grown in WT mice. Similarly, smaller and fewer lung metastatic lesion, developed in Gal-8-KO mice, when compared with metastatic lesions developed in their WT control littermates [70]. These results suggest that the lower levels of cytokines/chemokines expressed in Gal-8 KO mice may contribute to the reduced formation of primary tumors and metastatic lesions in these animals.

Additional mechanisms may contribute to the pro-metastatic action of Gal-8. These include the promotion of homotypic aggregation of the tumor cells as well as increased cell–matrix interactions that increase cell growth, adhesion, and selective metastatic seeding [37,38,104]. This can be attributed to the role of Gal-8 as an extracellular matrix protein, equipotent to fibronectin in promoting cell adhesion, spreading, and migration [10,12]. Accordingly, Gal-8 silencing inhibits filopodia formation [12], and aggregation of cancer cells [37]; processes that are actively engaged in metastatic progression. Based on the above findings it seems reasonable to speculate that Gal-8 inhibitors might turn useful in the treatment of at least certain tumor types. The reduced cytokine/expression and the consequent reduced immunity of patients undergoing such treatment should be taken into consideration, however, it should be weighted against the cytotoxic and cytistatic effects of other anti-cancer therapies.

## Effects of cytokines on galectin expression

Galectin expression is regulated by different stimuli. For example, Gal-1 up-regulation is associated with osteoarthritic cartilage and subchondral bone histopathology and severity of degeneration [116]. Intestinal epithelial cells (IECs) release immunomodulatory galectins upon exposure to CpG DNA (mimicking bacterial triggers) [117]. TGF-β1 triggers a Smad-dependent pathway to control Gal-1 expression in HL-60 cells [118] while extracellular stress stimuli trigger the expression of Gal-3 [119].

Much less is known about the direct effects of cytokines on galectin expression and secretion. The expression of Gal-9 is induced by IFN-γ and IL-1β in various cell types [120,121]. In contrast, TNF-α reduces Gal-3 expression in human OA and rheumatoid arthritis synovial fibroblasts [122]. Similarly, IL-1β and TNF-α decrease Gal-1 and Gal-3 gene expression in Equine bone-marrow-derived mesenchymal stromal cells (BMSCs) [123], suggesting that cytokines may have dual or even conflicting roles as regulators of galectin expression and secretion.

Up-regulation of endogenous Gal-8 expression upon inflammatory response has been reported, although the direct involvement of cytokines in this process is less clear. High levels of Gal-8 were found in the synovial fluid of rheumatoid arthritis (RA) patients [7] and in chondrocytes of osteoarthritis (OA) patients [86]. Gal-8 is markedly increased in endothelial cells surrounded by perivascular inflammatory infiltrates [52] and higher Gal-8 expression is observed in DCs and B cells upon activation of TLR-4 signaling [57]. Similarly, thrombin-treated human platelets [124], and LPS-activated endothelial cells express and secrete higher amounts of Gal-8 [85]. Of note, LPS stimulation induced secretion of the Gal-8M isoform, while the content of the Gal-8L isoform remained unchanged in culture supernatants [85]. The above findings indicate that pro-inflammatory conditions enhance Gal-8 expression and secretion under different settings. Furthermore, inflammation might affect the secretion of only a subset of the Gal-8 isoforms. Still, there is no detailed understanding of the mechanisms involved, and there is no evidence whether cytokines can directly induce Gal-8 expression in a cell-autonomous manner. Hence, this research area requires much further development.

## Direct galectin–cytokine interactions

Recent studies raise the interesting possibility that galectins and cytokines can associate as heterodimers with functional consequences [61,125]. In particular, Gal-3 secreted by tumors cells binds glycosylated IFNγ and IL-12, thus avoiding IFNγ diffusion and the formation of an IFNγ-induced chemokine gradient required for T cell infiltration [125]. Gal-1 and Gal-3 were shown to interact with cytokines and chemokines as evidenced by solid-phase immunoassays and surface plasmon resonance (SPR). Heterodimer formation between Gal-3 and SDF-1 were also documented. This novel type of interaction is an important addition to the known ability of galectins to form homodimers [126], as well as galectin/galectin heterodimers [127]. Functionally, binding of the Gal-3 CRD blocks SDF-1-mediated leukocyte migration. This blockade presumably involves the formation of ternary complex of SDF-1, its receptor CXCR4 and the Gal-3 CRD that inhibits CXCR4-mediated signaling without interfering with receptor internalization [61]. Further studies are still required to establish a potential involvement of Gal-8 in direct interactions with cytokines.

## Galectins and the ‘cytokine storm’

The major cause of fatality in COVID-19 infected patients, is referred as the ‘cytokine storm syndrome’ (CSS). It is a direct result of an aberrant immune activation that causes excess release of inflammatory cytokines by macrophages, monocytes, and dendritic cells [128]. Building upon the known functions of galectins as modulators of adaptive and innate immune responses [26,48] it is reasonable to assume a key role for galectins in the pathogenesis of COVID-19. Indeed, significantly elevated levels of Gal -1 -3, and -9 were reported in plasma of patients with severe COVID-19 [129–131]. Gal-1 represses innate and adaptive immune programs, while Gal-3 and -9 amplify inflammatory responses during sepsis and several types of infection. Therefore, it is reasonable to assume that Gal-3 and -9 are elevated in the early phases that promote cytokine storm, while increased levels of Gal-1 are presumably linked to a negative-feedback control mechanism, where the body attempts to dampen the vigorous immune response. The formation of galectin–cytokine heterodimers that attenuate cytokine signaling [61] might also play a role.

Glycan-mediated interactions are essential for the initial contact between many viruses and their hosts [132] and galectins directly affect viral–host interactions [133]. For example, Gal-3 binding to the viral protein UL-46 promotes HSV-1 infection to host cells [134]. Similarly, Gal-3 facilitates exosome-mediated viral infection by its interaction with membrane fibronectin [135] and by the creation of a biofilm that promotes viral adhesion to host cells [136]. The SARS-CoV-2 virus employs a glycosylated spike protein (S) to bind the angiotensin-converting enzyme 2 (ACE2) of the host [137,138]. Both ACE2 and the viral receptor-binding domain (RBD) are glycosylated, suggesting galectins as their potential binding partners. Indeed, recent studies employing NMR revealed that Gal-8N binds exclusively to the 3′SLacNAc RBD of SARS-CoV-2, whereas Gal-3 and Gal-7 recognize additional motifs of the RBD[139], but the functional consequences of such interactions are currently unclear. Combined with its potential to stimulate the expression and secretion of many pro-inflammatory cytokines, it is tempting to speculate that Gal-8, similar to Gal-3 [128,140], might affect the formation of a cytokine storm.

## Conclusion

**Figure 1.**
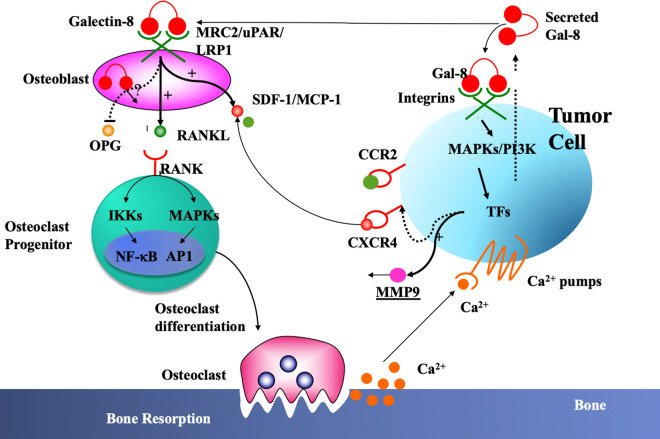
Role of Gal-8 in osteolytic bone Metastasis-Induction of a ‘Vicious cycle’. Dissemination of Gal-8, expressed by the primary tumor cells and by the tumor microenvironment induces in an autocrine and paracrine manner the expression and secretion of cytokines and chemokines at the primary tumor site that promotes primary tumor growth. In addition, extracellular gal-8, secreted at the metastatic niche further enhances the production of cytokines/chemokines that chemoattract cancer cells to this site. The role of intracellular Gal-8 in these processes still needs to be determined.

The presented studies suggest the existence of a ‘vicious cycle’ (Figure 1) whereby Gal-8, secreted by tumor and naïve cells present in the tumor microenvironment, promotes in an autocrine and paracrine manner the secretion of chemokines, cytokines, and additional proteins (e.g. MMP9, GAS6) that support tumor growth and induce recruitment of cancer cells to the metastatic niche. Gal-8 secretion by newly recruited cancer cells further fuels cytokine production and chemoattraction of cancer cells. The effects of Gal-8 on cytokine/chemokine expression seem to have a physiological significance since total-body Gal-8 KO mice [87] show reduced expression of cytokines and chemokines while the opposite is true for Gal-8-Tg mice [69,70].

**Figure 2.**
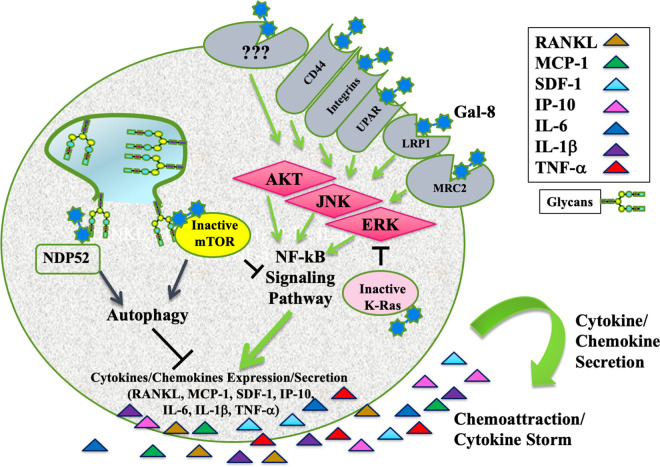
Effects of Gal-8 on cytokine expression and secretion. Intracellular Gal-8 mediates autophagy through binding to glycans of ruptured vacuolar membranes and the autophagy receptor NDP52 to initiate the formation of autophagosomes. Autophagy is considered as inhibitory to the action of inflammasomes that promote the generation of selected cytokines. Gal-8 binding to exposed luminal glycans of damaged lysosomal membranes inactivates mTOR. Direct *in vitro* interactions of intracellular Gal-8 with K-Ras, inhibit K-Ras activity and abrogates ERK signaling pathway. ERK and mTOR stimulate the NF-κB pathway, therefore, their inhibition by intracellular Gal-8 is expected to dampen cytokine/chemokine expression and secretion. Gal-8 also functions extracellularly. Extracellular Gal-8 binds to a complex of cell surface receptors that include LRP1, uPAR, and MRC2; CD44 and members of the integrin family. Their ligation triggers many signaling cascades including AKT, ERK, and JNK that stimulate the NFkB signaling pathways that converge upon cytokine/chemokine production and secretion. Activation of specific cytokines is presumably mediated by different signaling pathways. For example, RANKL expression in osteoblasts is mediated by the ERK pathway, whereas expression of SDF-1 in the same cells, is triggered by JNK. The secreted cytokines serve as chemo-attractants of cancer cells and as potential inducers of a ‘cytokine storm’. The interplay between the actions of intracellular vs. extracellular Gal-8 deserves further elucidation.

The underlying mechanism involves binding of Gal-8 to a complex of cell surface receptors that include LRP1, uPAR, and MRC2; activation of AKT, ERK, JNK, and NFkB signaling pathways; and induction of cytokine/chemokine production (Figure 2). Receptors such as CD44 [7] or members of the integrin family [9,10] are additional candidates to mediate the effects of Gal-8 on cytokine secretion as these receptors are binding partners of Gal-8 and were reported to trigger signaling pathways that converge upon activation of the NF-κB pathway [141–143]. Importantly, cytokine expression is mediated by different signaling pathways. For example, RANKL expression in osteoblasts is mediated by the ERK pathway [69], whereas expression of SDF-1 in the same cells, is triggered by JNK [70].

The apparent discrepancy between the action of intracellular vs. extracellular Gal-8 on cytokine expression and secretion deserves further attention. By promoting autophagy, intracellular Gal-8 inhibits activation of the NLRP3 inflammasome and the formation of cytokines. Similarly, intracellular Gal-8 exerts inhibitory ques to the Ras/ERK/mTOR signaling pathways, that are otherwise activated by extracellular Gal-8 to promote cytokine expression. Hence, further studies, mainly in animal models, are required to solve this apparent puzzle.

The systemic reduction in cytokine and chemokine expression renders Gal-8 KO animals partially resistant to the growth and development of primary tumors and metastatic lesions. This is in accord with the notion that cytokines and chemokines promote the growth of primary tumors, and support the recruitment of cancer cells to the metastatic niche [39]. The injected tumor cells express endogenous Gal-8, still, they form tumors of reduced size, when implanted into Gal-8 KO animals. Given that Gal-8 does not control the primary growth of cancer cells in a cell-autonomous manner [37], it is reasonable to assume that the tumor microenvironment, that consists of cells deficient in Gal-8 that expresses low levels of cytokines and chemokines, accounts for the reduced growth of the primary tumor. Hence, Gal-8 affects indirectly tumor growth, as a result of its action on the extent of secretion of cytokines by the tumor microenvironment.

Still, several studies reported on decreased expression of Gal-8 in association with favorable early tumor progression [100,108,144]. This suggests that Gal-8 exerts a delicate balance between its effects on cytokine/chemokine expression that promote cancer growth vs. its effects on cytokine-mediated immune responses that inhibit cancer progression. The ‘heavier arm’ of this delicate balance eventually dictates whether Gal-8 is beneficial or deleterious to tumor growth and metastasis. Finally, it should also be kept in mind that many studies described here make use of animal models that not always recapitulate human biology. Caution should therefore be exercised when attempting to translate these findings to humans.

A different angle emerges from understanding that a ‘cytokine storm’ underlies poor prognosis of COVID-19 patients [145]. Given that Gal-8 is a potent stimulator of cytokine expression, it might promote the ‘storm’ yet, its potential direct interactions with cytokines might offset its pro-inflammatory activity. Similarly, Gal-8 direct binding at the SARS-CoV-2 coronavirus RBD, might impede viral infection. Prototypes of Gal-8 inhibitors [146,147] are already available. Yet, further studies are required to unravel the role of Gal-8 in tumor growth and in the immunopathogenesis of COVID-19, before considering it as a potential therapeutic target.

## Perspectives

- *Importance of the field:* Galectins are key mediators of adaptive and innate immune responses and play central roles in immune regulatory cancer networks. Given the importance of cytokine and chemokine in these very same cellular responses and networks, it is highly relevant to explore the direct interplay and reciprocal systemic effects of galectins including Gal-8 on cytokine/chemokine expression and function mainly in non-immune cells; an important field that remains incompletely understood.
- *Current thinking:* The current studies suggest the existence of a ‘vicious cycle’ whereby Gal-8 expression and secretion promotes in an autocrine and paracrine manner secretion of cytokines and chemokines that further fuels Gal-8 expression. This ‘vicious cycle’ supports tumor growth and induces the recruitment of cancer cells to the metastatic niche. It could also enhance the development of a ‘cytokine storm’ which is a key contributor to the poor prognosis of COVID-19 patients.
- *Future directions:* Future studies are needed to reveal the underlying mechanism utilized by Gal-8 to promote cytokine and chemokine expression and secretion from non-immune cells. Even less studied are the reciprocal effects of cytokines and chemokines on the expression, secretion, and function of Gal-8. The direct interactions and complex formation between Gal-8 and individual cytokines/chemokines need unraveling, and the physiological consequences of these interactions needs to be revealed. Additional detailed studies are required to clarify the interplay between the action of intracellular vs. extracellular Gal-8 and their physiological role in the regulation of immune responses and cancer progression.

In the context of the COVID-19 pandemic, it is necessary to clarify the physiological balance between the action of Gal-8 as a promoter of cytokine secretion vs. its action as a direct binding partner of cytokines that might impede their activity. Gal-8 binding at the SARS-CoV-2 coronavirus RBD should be evaluated *in vivo* and its physiological consequences should be revealed. Collectively, further studies are required to unravel the importance of Gal-8 in the immunopathogenesis of COVID-19 and its possible consideration as potential therapeutic target.

## Open Access

Open access for this article was enabled by the participation of Weizmann Institute of Science in an all-inclusive Read & Publish pilot with Portland Press and the Biochemical Society under a transformative agreement with MALMAD.

## Abbreviations

ACE2: angiotensin-converting enzyme 2
CRD: carbohydrate-recognition domain
CSS: cytokine storm syndrome
Gal-8: galectin-8
KO: knockout
NF-κB: nuclear factor-κB
OA: osteoarthritis
RA: rheumatoid arthritis
RANKL: receptor activator of NF-κB ligand
RBD: receptor-binding domain
STING: stimulator of interferon genes
TLRs: Toll-like receptors
TNF-R: tumor necrosis factor
